# Para Além da Tradução: Construindo uma Aliança Lusófona para a Saúde Cardiovascular

**DOI:** 10.36660/abc.20260065

**Published:** 2026-03-03

**Authors:** Flávia Geraldes

**Affiliations:** 1 The Lancet Londres Inglaterra The Lancet, Londres – Inglaterra

**Keywords:** Doenças Cardiovasculares, Atenção Primária à Saúde, Sistemas de Saúde, Cooperação Internacional

A ciência da prevenção cardiovascular chegou a um platô de implementação. Apesar de um extenso arsenal de intervenções eficazes, incluindo farmacoterapia anti-hipertensiva, agentes hipolipemiantes, controle do tabagismo e redução de riscos a nível populacional, a carga global das doenças não comunicáveis (DNC) continua a aumentar, particularmente nos países de baixa e média renda.^1^ Mas a falha não está nas descobertas, e sim na implementação. O modelo predominante de geração de evidência em saúde cardiovascular, amplamente produzido, debatido e disseminado nos sistemas de alta renda e intensivos em pesquisa, tem enfrentado dificuldade em ganhar tração no contexto de outros sistemas de saúde para os quais as DNC são a principal causa de morbidade e mortalidade.

Intervenções validadas em ambientes com ampla disponibilidade de recursos e altamente especializados serão muitas vezes implementadas, se é que chegam a sê-lo,^2^ em sistemas que enfrentam escassez de força de trabalho, linhas de cuidado fragmentadas, e financiamento restrito, além de apresentarem diferentes perfis epidemiológicos.^3^ Como resultado, uma evidência robusta *em princípio* pode revelar-se frágil *na prática*. A superação dessa lacuna requer não apenas tradução linguística, mas também de sistemas.

Neste contexto, a Comunidade dos Países de Língua Portuguesa (CPLP) representa mais do que um artefato cultural ou histórico. Trata-se de plataforma subutilizada, mas estrategicamente significativa, com potencial para ajudar a enfrentar a crise de implementação na saúde cardiovascular.^4^ Totalizando cerca de 280 milhões de pessoas em quatro continentes, os países de língua portuguesa abrangem um amplo espectro de níveis de renda, de estruturas demográficas e de capacidade dos respetivos sistemas de saúde, mas enfrentam desafios convergentes no que diz respeito a DNC. A concretização do potencial desse espaço compartilhado requer ir além de apelos simbólicos à herança comum, e em direção a uma aliança deliberadamente operacional, fundamentada em evidência e infraestrutura compartilhada.^5^

A língua está no centro desse desafio, pois a implementação depende tanto de como a evidência é comunicada quanto da própria evidência. O inglês tornou-se a língua franca indispensável para a ciência moderna, possibilitando um intercâmbio global rápido, o compartilhamento de padrões metodológicos e a construção de conhecimento cumulativo em uma escala que seria de outra maneira impossível. Esse papel não deve ser contestado, mas a dependência quase exclusiva do inglês como língua de produção e debate científicos tem consequências frequentemente ignoradas, em particular quando o foco se desloca da descoberta para a implementação.^6^

As diretrizes e políticas cardiovasculares globais elaboradas predominantemente em inglês tendem inevitavelmente a refletir os pressupostos, os modelos organizacionais e a disponibilidade de recursos dos sistemas em que foram produzidos. Para um agente comunitário de saúde na área rural de Moçambique, uma enfermeira que lida com doença crônica em uma clínica municipal no Brasil ou um clínico geral no Timor-Leste, a “evidência disponível” pode existir em teoria, mas permanecer inutilizável na linha de frente do cuidado. Recomendações clínicas concebidas para sistemas com alto grau de especialização não se traduzem automaticamente em contextos em que a continuidade do cuidado, o acesso ao diagnóstico e a composição da força de trabalho diferem fundamentalmente.^7^

A publicação de ciência cardiovascular de alta qualidade em português não é, portanto, um retrocesso em relação à excelência global, mas um pré-requisito para o impacto local. Permite que a evidência seja contextualizada, debatida e adaptada dentro dos ambientes profissionais e institucionais em que o cuidado é realmente prestado. Assim, pode-se reduzir a lacuna entre conhecimento e prática.^8^ Para que a medicina baseada em evidência sobreviva ao trajeto de um centro de pesquisa em Londres até uma clínica de atenção primária em Luanda, precisa ser traduzida não apenas linguisticamente, mas também estruturalmente.

Para além da disseminação, a aliança lusófona desafia a suposição ultrapassada de que a inovação flui em uma só direção: tipicamente, dos sistemas mais ricos para aqueles com menos recursos. Na prática, algumas das lições mais relevantes para a prevenção cardiovascular emergem de contextos que foram obrigados a priorizar eficiência, continuidade e cuidados comunitários.

O Sistema Único de Saúde (SUS) no Brasil é um excelente exemplo. Através da sua Estratégia de Saúde da Família,^9^ o SUS construiu uma das maiores redes de atenção primária do mundo, ancorada em equipes multidisciplinares e agentes comunitários de saúde responsáveis por populações geograficamente definidas. É importante notar que, nesse modelo, o acompanhamento rotineiro dos pacientes com hipertensão e diabetes é amplamente realizado por agentes comunitários de saúde e enfermeiros, enquanto aos médicos cabe a supervisão clínica e o encaminhamento, e não o atendimento contínuo na linha de frente. Essa organização do cuidado favorece a adesão ao tratamento, a identificação precoce de complicações e o acompanhamento domiciliar de longo prazo. Para países como Angola ou Moçambique, nos quais a densidade de médicos permanece baixa (aproximadamente 2 médicos por 10 mil habitantes, em comparação com 24 no Brasil e 59 em Portugal),^10^ a lógica operacional do cuidado cardiovascular baseado em uma equipe e ancorado na comunidade, em vez de no serviço prestado primordialmente pelo médico, é diretamente transferível.

Por outro lado, o Brasil pode aprender a partir de outros contextos lusófonos. A experiência de Portugal ao lidar com o envelhecimento populacional^11^ reflete um estágio mais avançado da transição demográfica que o Brasil já enfrenta. Altos níveis de multimorbidade e doença cardiovascular entre pessoas idosas impõem demandas contínuas ao sistema de saúde português, reforçando a centralidade da atenção primária de saúde, da coordenação entre os níveis de cuidado e da continuidade da gestão clínica das condições cardiovasculares crônicas. Esses desafios e as respostas sistêmicas que eles exigem são cada vez mais relevantes para o Brasil à medida que a longevidade aumenta e a carga de doença crônica complexa cresce.

Os países de língua portuguesa na África constituem uma fonte adicional de inovação. Em cenários marcados pela escassez da força de trabalho, a delegação de tarefas expandiu-se do monitoramento para a tomada de decisão clínica. O manejo de hipertensão e risco cardiovascular por enfermeiros e nos serviços comunitários de saúde demonstrou como a continuidade do cuidado pode ser mantida apesar da disponibilidade limitada de especialistas.^12^ Tais modelos oferecem valiosas lições para melhorar o acesso ao cuidado no vasto interior do Brasil, onde atribuições clínicas permanecem com frequência restritas aos médicos, apesar de sua ausência. Tais exemplos mostram que o espaço lusófono não é uma hierarquia, mas uma rede, na qual o conhecimento circula de maneira horizontal e o contexto importa tanto quanto o nível de renda.

No entanto, para que a aliança lusófona na saúde cardiovascular tenha um impacto real, esse intercâmbio precisa ser operacionalizado. Intercâmbio informal e cooperação bilateral, ainda que valiosos, são insuficientes para sustentar um progresso duradouro. Faz-se necessária a construção de infraestrutura compartilhada, que permita a dados, mercados e pessoas funcionar como um sistema integrado e não como componentes isolados.

O alicerce de tal sistema são os dados. A ausência de vigilância cardiovascular harmonizada, um desafio documentado em diversos sistemas de saúde no mundo, limita tanto a tomada de decisão nacional quanto o aprendizado coletivo. Diferenças nas definições de doença, práticas de codificação e medidas de desfecho tornam difícil uma comparação significativa, mesmo em locais com desafios epidemiológicos semelhantes.^13^ Um registro cardiovascular lusófono unificado, construído com base em definições de dados padronizados e plataformas interoperáveis, transformaria esse panorama. Alinhando a coleta de dados nos países da CPLP, seria possível gerar conhecimentos epidemiológicos com uma escala e diversidade inalcançáveis por qualquer nação atuando de forma isolada.

Dados comparáveis permitem pesquisas de efetividade e uma avaliação mais detalhada do desempenho das intervenções em diferentes sistemas de saúde. Seria possível, por exemplo, examinar a variação no controle da pressão arterial ou adesão ao tratamento nas diferentes populações urbanas e rurais em Luanda, Lisboa e Salvador, gerando evidências diretamente aplicáveis à prática.

A continuidade dos dados, por sua vez, gera poder de negociação nos mercados. Muitos países de língua portuguesa enfrentam desafios similares em assegurar acesso regular e sustentável a medicamentos essenciais para a prevenção e o tratamento das DNC. Aquisições descentralizadas e não coordenadas, um limitado poder de negociação e capacidades regulatórias variáveis contribuem para desigualdades nos preços e inconsistência no fornecimento.^14^ Estratégias de aquisição coordenadas, seja por meio de compras conjuntas, listas padronizadas de medicamentos ou negociações de preço alinhadas, poderiam melhorar substancialmente o acesso. A aquisição em grande escala de medicamentos cardiovasculares amplamente utilizados, como estatinas ou análogos da insulina, por um bloco que reúne centenas de milhões de pessoas representa uma posição de negociação fundamentalmente diferente daquela de ministérios individuais agindo individualmente.

Em última análise, essa infraestrutura depende de pessoas. Um ecossistema compartilhado de dados e aquisições requer uma força de trabalho capaz de atuar em diferentes contextos de recursos e traduzir evidência em prática. Facilitar a mobilidade educacional dentro do mundo lusófono, através do reconhecimento mútuo da formação, do compartilhamento de currículos e de estágios de aperfeiçoamento transfronteiriços, poderia contribuir para o desenvolvimento dessa capacidade. Clínicos e pesquisadores formados em múltiplos contextos de lusófonos estariam particularmente bem posicionados para navegar diferenças de recursos, regulamentações e perfis populacionais, fortalecendo a implementação em larga escala. Para que essa capacidade se sustente, a mobilidade educacional e a integração da força de trabalho precisam ser amparadas por compromisso governamental e incorporadas em políticas públicas mais amplas, viabilizadas não apenas pela língua, mas também por compatibilidades administrativas e jurídicas. Muitos países lusófonos compartilham tradições de direito civil e marcos regulatórios semelhantes,^15^ o que pode reduzir as barreiras ao alinhamento dos processos de revisão ética, à acreditação profissional e aos modelos de aquisição. Essa interoperabilidade institucional torna a colaboração prática mais viável do que frequentemente se supõe.

Por fim, os periódicos médicos ocupam uma posição decisiva na definição do êxito dessa transição. Se os periódicos de língua portuguesa forem tratados apenas como canais secundários para trabalhos que não encontraram espaço em outros meios, reforça-se um ciclo de marginalização. Em contrapartida, se forem posicionados como espaços centrais para a ciência da implementação — o ‘como fazer’ da prevenção e do cuidado cardiovascular — tais periódicos podem acelerar a tradução de evidência em impacto. Esses periódicos podem promover o diálogo entre os países, legitimar questões de pesquisa contextualmente relevantes e oferecer uma plataforma para análises comparativas que, de outra forma, teriam pouca visibilidade. Ao selecionar trabalhos de alta qualidade que dialoguem diretamente com as realidades dos sistemas de saúde de língua portuguesa, esses periódicos podem fortalecer a relevância de políticas públicas e o engajamento profissional ao mesmo tempo, mantendo-se plenamente alinhados aos padrões científicos globais.

A carga das DNC constitui o principal desafio de saúde pública do nosso século. Enfrentá-la de forma isolada — seja no âmbito de países individuais, seja no de periódicos isolados — é garantia de impacto limitado. No mundo lusófono, a língua comum oferece uma base prática para a colaboração transcontinental, não porque o português seja intrinsecamente mais adequado para a ciência, mas porque já conecta países por meio de redes institucionais, legais e profissionais consolidadas. A oportunidade agora é utilizar deliberadamente essa língua comum como instrumento de implementação, equidade e ação coletiva — em vez de tratá-la como uma herança passiva do passado.
