# Supplementary material for: Para Além da Tradução: Construindo uma Aliança Lusófona para a Saúde Cardiovascular
Source: Arq Bras Cardiol. 2026 Mar 3;123(2):e20260065. [Article in Portuguese] doi: 10.36660/abc.20260065 (PMC13148859; doi:10.36660/abc.20260065)
Supplement: Translation

# Beyond Translation: Building a Lusophone Alliance for Cardiovascular Care

**DOI:** 10.36660/abc.20260065i

**Authors:** Flávia Geraldes

**Affiliations:** 1 England The Lancet, London – England

**Keywords:** Cardiovascular Diseases, Primary Health Care, Health Systems, International Cooperation

The science of cardiovascular prevention has reached a plateau of implementation. Despite an extensive arsenal of effective interventions, including antihypertensive pharmacotherapy, lipid-lowering agents, tobacco control, and population-level risk reduction, the global burden of non-communicable diseases (NCDs) continues to rise, particularly in low-income and middle-income countries.^1^ But the failure is not one of discovery; it is of delivery. The prevailing model of cardiovascular evidence generation—largely produced, debated, and disseminated within high-income, research-intensive systems—has struggled to gain traction in other health-system contexts where NCDs account for the greatest share of morbidity and mortality.

Interventions validated in well-resourced, highly specialised environments will often be implemented, if at all,^2^ within systems challenged by workforce shortages, fragmented care pathways, constrained financing, and with different epidemiological profiles.^3^ As a result, evidence that is robust in *principle* may prove fragile in *practice*. Bridging this gap requires not just translation of language, but also of systems.

In this context, the Community of Portuguese Language Countries (CPLP) represents more than a cultural or historical artifact. It is a dormant but strategically significant platform that can help to address the implementation crisis in cardiovascular health.^4^ Encompassing around 280 million people across four continents, Portuguese-speaking countries span a wide spectrum of income levels, demographic structures, and health-system capacities—yet face overlapping NCD challenges. Realising the potential of this shared space requires a move beyond symbolic appeals to common heritage and towards a deliberately operational alliance, grounded in evidence and shared infrastructure.^5^

Language sits at the centre of this challenge, because implementation depends as much on how evidence is communicated as on what evidence exists. English has become the indispensable *lingua franca* of modern science, enabling rapid global exchange, shared methodological standards, and cumulative knowledge-building at a scale that would otherwise be impossible. This role should not be contested, but near-exclusive reliance on English as the language of scientific production and debate has consequences that are often overlooked, particularly when the objective shifts from discovery to implementation.^6^

Global cardiovascular guidelines and policy frameworks developed primarily in English tend, unavoidably, to reflect the assumptions, organisational models, and resource environments of the systems in which they are produced. For a community health worker in rural Mozambique, a nurse managing chronic disease in a municipal clinic in Brazil, or a general practitioner in Timor-Leste, “available evidence” may exist in theory, yet remain unusable at the point of care. Clinical recommendations framed for highly specialised systems do not automatically translate into settings where continuity of care, diagnostic access, and workforce composition differ fundamentally.^7^

Publishing high-quality cardiovascular science in Portuguese is therefore not a retreat from global excellence. It is a prerequisite for local impact. It enables evidence to be contextualised, debated, and adapted within the professional and institutional environments where care is actually delivered. Doing so can help bridge the persistent “know–do” gap.^8^ If evidence-based medicine is to survive the journey from a trial site in London to a primary care clinic in Luanda, it must be translated not only linguistically, but structurally.

Beyond dissemination, a Lusophone alliance also challenges the outdated assumption that innovation flows in a single direction: typically, from wealthier systems to those with fewer resources. In practice, some of the most relevant lessons for cardiovascular prevention emerge from contexts that have been forced to prioritise efficiency, continuity, and community-based care.

Brazil’s *Sistema Único de Saúde* (SUS) offers a prominent example. Through its Family Health Strategy,^9^ SUS has built one of the world’s largest primary care networks, anchored by multidisciplinary teams and community health agents responsible for defined geographic populations. Crucially, routine follow-up of patients with hypertension and diabetes is delivered largely by community health agents and nurses, with physicians providing supervisory and referral support rather than continuous front-line care, supporting medication adherence, early identification of complications, and longitudinal household-level care. For countries such as Angola or Mozambique, where physician density remains low (approximately 2 medical doctors per 10,000 population; compared with 24 in Brazil and 59 in Portugal),^10^ the operational logic of team-based, community-anchored cardiovascular care, rather than physician-intensive service delivery, is directly transferable.

Conversely, Brazil itself stands to learn from other Lusophone contexts. Portugal’s experience responding to population ageing^11^ reflects a more advanced stage of the demographic transition Brazil is already undergoing. High levels of multimorbidity and cardiovascular disease among older adults have placed sustained demands on the Portuguese health system, reinforcing the centrality of primary care, coordination across care levels, and continuity in the management of chronic cardiovascular conditions. These challenges, and the system responses they require, are increasingly relevant for Brazil as longevity rises and the burden of complex chronic disease grows.

Portuguese-speaking countries in Africa provide a further source of innovation. In settings constrained by workforce scarcity, task-shifting has expanded beyond monitoring to clinical decision-making. Nurse-led and community-based management of hypertension and cardiovascular risk have demonstrated how continuity of care can be maintained despite limited specialist availability.^12^ Such models offer valuable lessons for improving access in Brazil’s vast interior, where clinical duties often remain restricted to physicians despite their absence. These examples illustrate that the Lusophone space is not a hierarchy but a network, in which expertise circulates horizontally and context matters as much as income level.

For a Lusophone alliance in cardiovascular health to carry real weight, however, this exchange must be operationalised. Informal exchange and bilateral cooperation, while valuable, are insufficient to support sustained progress. What is required is the construction of shared infrastructure that allows data, markets, and people to function as an integrated system rather than as isolated components.

The foundation of such a system is data. The absence of harmonised cardiovascular surveillance—a challenge documented across many health systems globally—limits both national decision-making and collective learning. Differences in disease definitions, coding practices, and outcome measures make meaningful comparison difficult, even where epidemiological challenges are similar.^13^ A unified Lusophone cardiovascular registry, built on standardised data definitions and interoperable platforms, would transform this landscape. By aligning data collection across CPLP countries, it would become possible to generate epidemiological insights with a scale and diversity that no single nation could achieve alone.

Comparable data enable effectiveness research and more nuanced assessment of how interventions perform across health systems. One could, for example, examine variation in blood pressure control or treatment adherence across urban and rural populations in Luanda, Lisbon, and Salvador, generating implementation-relevant evidence that directly informs practice.

Data continuity, in turn, creates leverage in markets. Many Portuguese-speaking countries face similar challenges in securing reliable access to essential medicines for NCD prevention and treatment. Fragmented procurement, limited negotiating power, and variable regulatory capacity contribute to price disparities and inconsistent supply.^14^ Coordinated procurement strategies, whether through joint purchasing, shared formularies, or aligned price negotiations, could substantially improve access. The bulk procurement of widely used cardiovascular medicines such as statins or insulin analogues by a bloc representing hundreds of millions of people represents a fundamentally different negotiating position from that of individual ministries acting alone.

Ultimately, sustaining such infrastructure depends on people. A shared data and procurement ecosystem requires a workforce capable of operating across diverse resource settings and translating evidence into practice. Facilitating educational mobility within the Lusophone world, through mutual recognition of training, shared curricula, and cross-border fellowships, could help cultivate such capacity. Clinicians and researchers trained across multiple Portuguese-speaking contexts would be uniquely positioned to navigate differences in resources, regulations, and patient populations, strengthening implementation at scale. For such capacity to be sustained, educational mobility and workforce integration must be supported by government commitment and embedded within broader public policy frameworks, facilitated not only by language but also by administrative and legal compatibility. Many Lusophone countries share civil-law traditions and similar regulatory architectures,^15^ which can lower the barriers to aligning ethical review processes, professional accreditation, and procurement frameworks. This institutional interoperability makes practical collaboration more feasible than is often assumed.

Finally, medical journals occupy a pivotal position in determining whether this transition succeeds. If Portuguese-language journals are treated merely as secondary outlets for work that failed to find space elsewhere, a cycle of marginalisation is reinforced. But if they are instead positioned as primary venues for implementation science—the “how-to” of cardiovascular prevention and care—they can accelerate translation from evidence to impact. Journals can convene cross-country dialogue, legitimise context-specific research questions, and provide a platform for comparative analyses that would otherwise struggle for visibility. By curating high-quality work that speaks directly to the realities of Portuguese-speaking health systems, they can strengthen policy relevance and professional engagement while remaining firmly embedded in global scientific standards.

The burden of NCDs is the defining health challenge of our century. Confronting it in isolation, whether at the level of individual countries or individual journals, is a guarantee of limited impact. In the Lusophone world, a shared language offers a practical foundation for transcontinental collaboration, not because Portuguese is uniquely suited to science, but because it already links countries within established institutional, legal, and professional networks. The opportunity now is to use that shared language deliberately—as a tool for implementation, equity, and collective action—rather than treating it as a passive inheritance of the past.
